# Associations between proinflammatory cytokines in the synovial fluid and radiographic grading and pain-related scores in 47 consecutive patients with osteoarthritis of the knee

**DOI:** 10.1186/1471-2474-12-144

**Published:** 2011-06-30

**Authors:** Sumihisa Orita, Takana Koshi, Takeshi Mitsuka, Masayuki Miyagi, Gen Inoue, Gen Arai, Tetsuhiro Ishikawa, Eiji Hanaoka, Keishi Yamashita, Masaomi Yamashita, Yawara Eguchi, Tomoaki Toyone, Kazuhisa Takahashi, Seiji Ohtori

**Affiliations:** 1 Department of Orthopaedic Surgery, Chiba Rosai Hospital, Chiba, Japan; 2 Department of Orthopaedic Surgery, Seirei Sakura Citizen Hospital, Chiba, Japan; 3 Department of Orthopaedic Surgery, Social Insurance Funabashi Central Hospital, Chiba, Japan; 4 Department of Orthopaedic Surgery, Graduate School of Medicine, Chiba University, Chiba, Japan; 5 Department of Orthopaedic Surgery, Chiba Social Insurance Hospital, Chiba, Japan; 6 Department of Orthopaedic Surgery, Teikyo University Chiba Medical Center, Chiba, Japan

## Abstract

**Background:**

One of the sources of knee pain in osteoarthritis (OA) is believed to be related to local chronic inflammation of the knee joints, which involves the production of inflammatory cytokines such as tumor necrosis factor alpha (TNFα), interleukin (IL)-6, and nerve growth factor (NGF) in the synovial membrane, and these cytokines are believed to promote pathological OA. In the present study, correlations between proinflammatory cytokines in knee synovial fluid and radiographic changes and functional scores and pain scores among OA patients were examined.

**Methods:**

Synovial fluid was harvested from the knees of 47 consecutive OA patients, and the levels of TNFα, IL-6, and NGF were measured using enzyme-linked immunosorbent assays. Osteoarthritic knees were classified using Kellgren-Lawrence (KL) grading (1-4). The Western Ontario and McMaster University Osteoarthritis Index (WOMAC) was used to assess self-reported physical function, pain, and stiffness.

**Results:**

TNFα and IL-6 were detectable in knee synovial, whereas NGF was not. TNFα was not correlated with the KL grade, whereas IL-6 had a significantly negative correlation. We observed differences in the correlations between TNFα and IL-6 with WOMAC scores and their subscales (pain, stiffness, and physical function). TNFα exhibited a significant correlation with the total score and its 3 subscales, whereas IL-6 exhibited a moderately significant negative correlation only with the subscale of stiffness.

**Conclusions:**

The present study demonstrated that the concentrations of proinflammatory cytokines are correlated with KL grades and WOMAC scores in patients with knee OA. Although TNFα did not have a significant correlation with the radiographic grading, it was significantly associated with the WOMAC score. IL-6 had a significant negative correlation with the KL grading, whereas it had only a weakly significant correlation with the subscore of stiffness. The results suggest that these cytokines play a role in the pathogenesis of synovitis in osteoarthritic knees in different ways: TNFα is correlated with pain, whereas IL-6 is correlated with joint function.

## Background

Knee osteoarthritis (OA) is a common chronic degenerative disease characterized by the loss of articular cartilage components due to an imbalance between extracellular matrix destruction and repair [1]. The entire joint structure is affected, including the synovial membrane and subchondral bone, and OA can be recognized as an irregularity and deformity of joint spaces in radiographic images. Its main clinical sign is joint pain, which not only contributes to functional limitations and reduced quality of life but is also the leading cause of impaired mobility in the elderly population [2]. Although the exact mechanism of knee pain in OA is unclear, it is believed to be related to local chronic inflammation of the knee joints, which involves the production of inflammatory cytokines in the synovial membrane, such as tumor necrosis factor alpha (TNFα), interleukin (IL)-6, and nerve growth factor (NGF), which are generally considered to promote pathological OA [3-5]. Proinflammatory cytokine mediators have been reported to contribute to OA pathogenesis by increasing cartilage degradation and inducing hyperalgesia via a number of direct and indirect actions. TNFα activates sensory neurons directly via its receptors and initiates a cascade of inflammatory reactions via the production of ILs [6,7]. IL-6 is reported to have a complex role in OA pathogenesis by initiating inflammatory responses such as the production of tissue inhibitors of metalloproteinase, and this may act to limit cartilage damage via negative feedback [8]. NGF is reportedly upregulated in human osteoarthritic chondrocytes and synovial fibroblasts, suggesting its important role in the pathology of OA [6,9]. Another report indicated that NGF antagonism is an important mediator of pain in OA because its antagonistic effect resulted in analgesia in a murine OA model [10].

Thus, investigations of the dynamic states of these cytokines should be conducted. Additionally, a previous study indicated a strong association between the radiographic images of knees with OA and with knee pain [11]. Under the hypothesis that relationships between these cytokines and clinical evaluations in OA patients are possible, the present study evaluated the association between proinflammatory cytokines in the synovial fluid from the knees of OA patients and radiographic severity and pain scale scores.

## Methods

Our Institutional Review Board approved the present study. We obtained informed consent from each participating patient.

### Patient selection

The present study included adult patients with knee pain who visited our facility for clinical consultation between August 2009 and March 2010. The present study consisted of patients with knee OA diagnosed using the American College of Rheumatology criteria for OA who had not received any prior treatment. Patients with clear clinical evidence of any involvement of trauma, prior treatment, or other orthopedic diseases including spinal disorders causing radicular pain in the legs were excluded. Patients diagnosed with rheumatoid arthritis based on physical examination and laboratory data were also excluded.

### Synovial fluid sampling and cytokine assay

With the approval of patients, samples of synovial joint fluid were collected using a syringe and needle in our outpatient clinics by experienced orthopedic physicians. The samples of synovial fluid were aspirated directly without lavage and immediately stored at -70°C until use. Freeze-thaw cycles were avoided. Cytokine quantification was performed using a double-antibody sandwich enzyme-linked immunosorbent assay (ELISA) for TNFα, IL-6 (R&D systems, Minneapolis, MN), and NGF (Boster Biological Tec., Wuhan, China) without dilution according to the manufacturers' protocols (centrifugation before use: for 15 min at 1,000 × *g *(TNFα and IL-6) or for 20 min at 2,000 × *g *(NGF)). The detection limits of the assays were 0.5 pg/ml for TNFα, <0.70 pg/ml for IL-6, and <1 pg/ml for NGF. All samples were assessed in duplicate.

### Grading of OA and pain evaluation

Anteroposterior radiographs of the symptomatic knees were obtained. The X-ray beam was aimed at the lower pole of the patella and kept parallel to the joint surface. Radiographs were scored by experienced orthopedic surgeons using the Kellgren-Lawrence (KL) grading scale as follows: grade 1, doubtful narrowing of joint space and possible osteophytic lipping; grade 2, definite osteophytes and possible narrowing of joint space; grade 3, moderate multiple osteophytes, definite narrowing of joints space, some sclerosis, and possible deformity of bone contours; and grade 4, large osteophytes, marked narrowing of joint space, severe sclerosis, and definite deformity of bone contours [12]. The functional status and pain level of each patient were evaluated using the Western Ontario McMaster University Osteoarthritis Index (WOMAC) score [13]. The index consists of 3 subscales: pain, stiffness, and physical function. A higher score on the WOMAC scale represents poorer function or greater pain. The data were arranged according to the KL grade for each cytokine, and the correlations between the cytokines were analyzed. Correlations between the cytokine concentrations and WOMAC score were also analyzed.

### Statistical analysis

Statistical differences between the 2 groups were determined using the Mann-Whitney *U *test followed by Bonferroni's correction for multiple testing, and the statistical significance among the groups was determined using the Kruskal-Wallis test. The significance of correlations was determined by Spearman's rank correlation test (PASW statistics ver. 18 (SPSS Inc (IBM), Somers, NY)). A *p *value < 0.05 was considered significant.

## Results

### Patient demographics

Table 1 shows the patient demographics. Among the 50 patients enrolled in the present study, we could not obtain any fluid from the knees of 3 patients with osteoarthritic knees classified as KL grade 4, and thus, we analyzed the other 47 samples. Disease duration increased as the KL scores increased.

**Table 1:** Patient Demographics

	Gender		Disease duration (months)**	Total
			
	Male	Female		
No. of Patients	22 (21)	28 (26)		50 (47)
Average age (years)	69.6 ± 7.3	69.8 ± 11.3		70.0 ± 2.1
KL grading				
1	6	4	4.83 ± 2.0	10
2	6	9	14.8 ± 8.9	15
3	5	8	33.9 ± 18.6	13
4	5 (4)*	7 (5)*	45.3 ± 21.4	12 (9)*

*No fluid was obtained from 3 patients (1 man and 2 women), and thus, the numbers in parenthesis indicate the effective numbers for the present study.

**Described as mean ± SEM

### Concentrations of the proinflammatory cytokines

**Figure 1:**
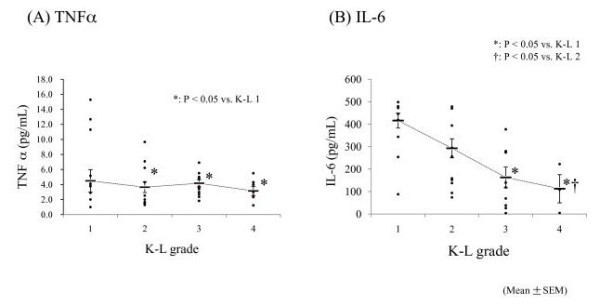
**Concentrations of proinflammatory cytokines in the synovial fluid from the knee joints**. (A) The concentration of TNFα exhibited no significant correlation with KL grading, although there was a tendency for increased TNFα concentrations at lower KL grades. (B) The concentration of IL-6 was significantly decreased in KL grades 3 and 4 compared with those in KL grades 1 and 2. NGF was undetectable in all patient samples.

Figure 1 shows the concentrations of proinflammatory cytokines in the synovial fluid of knee joints in relation to the radiographic findings for these joints. Measurable levels of TNFα and IL-6 were detected in all samples, whereas NGF was not detectable in any of the samples (CV value (%): TNFα, 5.8 ± 1.2; IL-6, 4.2 ± 0.18; NGF: unable to be calculated).

The concentration of TNFα was significantly lower in KL grades 2 to 4 than in KL grade 1 (KL 1, 6.5 ± 2.0 pg/ml (mean ± S.E.); KL 2, 3.6 ± 0.72 pg/ml (*p *= 0.38 vs. KL 1); KL 3, 4.2 ± 0.48 pg/ml (*p *= 0.025 vs. KL 1); and KL 4, 3.2 ± 0.86 pg/ml (*p *= 0.031 vs. KL 1)) (Figure 1A). The IL-6 concentration was significantly lower in KL grades 3 and 4 than in KL grades 1 and 2 (KL 1, 401.6 ± 33.2 pg/ml; KL 2, 292.6 ± 42.2 pg/ml; KL 3, 162.9 ± 46.5 pg/ml; and KL 4, 78.6 ± 62.8 pg/ml) (*p *= 0.032 vs. KL 1; *p *= 0.036 vs. KL 2) (Figure 1B). NGF was not detected in any sample.

### Correlation between WOMAC score and cytokine concentration

**Figure 2:**
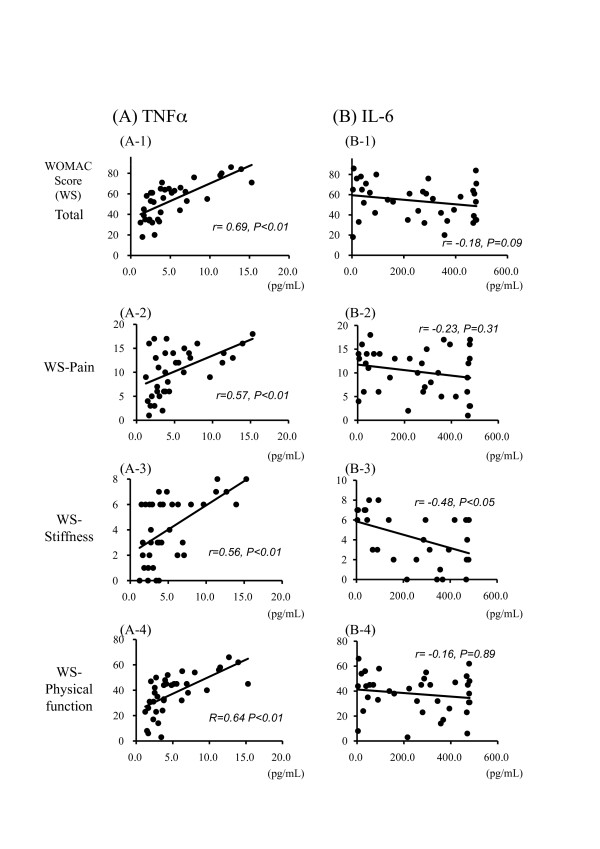
**Correlations between the detectable cytokines and the WOMAC score**. Group A shows TNFα, and group B shows IL-6. TNFα exhibited a moderately significant positive correlation with the total WOMAC score (A-1) and with the each subscale (pain (A-2), stiffness (A-3), and physical function (A-4) (*p *< 0.01)). IL-6 exhibited a weakly significant negative correlation only with stiffness (B-3) (*p *< 0.05), whereas it exhibited no correlation with the other subscales.

Figure 2 shows the correlations between the detectable cytokines and the WOMAC score. Group A shows TNFα, and group B shows IL-6. TNFα exhibited a moderately significant positive correlation with the total WOMAC score (A-1) and with each subscale (pain (A-2), stiffness (A-3), and physical function (A-4) (*p *< 0.01)). IL-6 exhibited a moderately significant negative correlation only with stiffness (B-3) (*p *< 0.05), whereas it did not exhibit any significant correlation with the other factors. The precise statistical values are shown in Table 2.

**Table 2:** Statistical data of Figure 2

	TNFα		IL-6	
	
	*r*	*p*	*r*	*p*
Total	0.69	0.0023	-0.18	0.09
Pain	0.57	0.0069	-0.23	0.31
Stiffness	0.56	0.0054	-0.48	0.039
Physical function	0.64	0.0038	-0.16	0.89

## Discussion

The present study examined whether inflammation plays a substantial role in the development of pain in OA. We demonstrated that TNFα and IL-6 were detectable in the synovial fluid sampled from the knees of OA patients, whereas NGF was undetectable. TNFα was not correlated with the KL grade, and IL-6 had a relatively significant negative correlation with KL grading. Some differences were found between TNFα and IL-6 regarding their correlations with the WOMAC score and its subscales. TNFα exhibited a moderately significant correlation with the total score and its 3 subscales, whereas IL-6 exhibited a weakly significant negative correlation with the subscale of stiffness. The WOMAC scoring method used in the present study was translated from the English version, and thus, we believe that its validity is similar as that reported in a previous study in Asian OA patients [14].

### Evidence of proinflammatory cytokines in the synovial fluid samples

The results of the present study are comparable with those of previous studies, which using a zymosan-induced mouse OA model, reported that TNFα is related to synovitis and that IL-6 has a role in reducing cartilage destruction [15-18]. These studies add importance to the present findings that TNFα inhibition may improve the WOMAC score and that increased IL-6 activity in earlier phases of OA prevents cartilage destruction. Brenner et al performed a similar experiment using the synovial membranes and fluid from OA patients, and reported that TNFα was undetectable in their synovial fluid and that there were no correlations between IL-6 levels and WOMAC pain subscores [19]. Regarding TNFα, some controversy exists among studies. Some previous studies reported low levels of TNFα in the synovial fluid of OA patients [20-22], whereas other studies including experiment models reported its detection [23-25]. The present study detected TNFα in the synovial fluid. These discrepancies may be attributable to the extremely low value of TNFα and the method of collecting and processing synovial fluid. However, we can suggest that TNFα is related to OA pathology and clinical evaluations based on the present findings, although additional investigations with greater numbers of samples are needed. Additionally, TNFα was also reported to not be regulated in the joints in late OA, and this is consistent with the finding of the present study [26].

As described in the Background section, NGF is considered an important factor in OA pathogenesis, and thus, it is important to discuss why NGF was not detected in the present study. We hypothesize that, excluding any technical errors, NGF production is insufficient for detection in the synovial fluid obtained from the knees of OA patients. According to a previous report, the mRNA expression of neurotrophins including NGF and its receptors was confirmed in the synovial fluid and tissues of patients with OA, whereas NGF mRNA expression was low [27]. Furthermore, NGF is a basic protein, and this is disadvantageous for its existence in the relatively acidic milieu of the synovial fluid [28]. Another study reported the diagnostic usefulness of biopsied tissue as opposed to the use of synovial fluid [26]. Thus, in addition to examining the synovial fluid, it may be important to investigate NGF expression in the subchondral tissue where inflammatory cytokines are reported to be produced [29]. Moreover, to investigate any correlation between proinflammatory cytokines including NGF, multivariable analysis among these cytokines should be performed in the future. Evaluating the levels of these cytokines in the synovial fluid from completely normal knees is important but also difficult for ethical reasons. Alternatively, we can evaluate the cytokine levels in the synovial fluid from injured knees such as those with anterior cruciate ligament (ACL) injuries; however, the data may not be useful because proinflammatory cytokine levels are elevated in response to any degradation or injury in the joint. However, we can partly infer their levels from previous studies. One study evaluating the cytokine levels in the knees of patients with chronic ACL deficiencies reported that TNFα concentrations were lower in injured knees than in normal knees, and the TNFα levels reported in that study were also low compared with those in the present study [30]. Because IL-6 and TNFα levels are elevated in the early phase of knee injuries, it will be important to measure their levels in normal knees.

### Correlation between cytokine concentrations and KL grading

A previous study indicated that the levels of TNFα in the synovial fluid were positively correlated with Larsen's radiographic grading of bone destruction in rheumatoid arthritis patients, whereas no correlation between the concentration of TNFα and Dahlgren's radiographic OA grade was found [31]. The report is consistent with some of the results of the present study regarding TNFα but not IL-6. The present study revealed that TNFα is clearly not correlated with joint degeneration as assessed by KL grading, whereas IL-6 is negatively correlated with KL grading, suggesting that IL-6 has an important role in OA progression. Generally, members of the IL family are reported to be related to the severity of cartilage destruction [32,33] The present study indicated that IL-6 production might be increased in the early stage of joint destruction in OA patients. Other studies have reported that TNFα induces IL-6 upregulation, and thus, IL-6 may still be correlated with OA progression [34-36]. Increased IL-6 activity has been reported to be associated with increased proteoglycan synthesis in articular cartilage in dogs with experimental ACL transaction [24], and thus, IL-6 production is highest in the early stages of joint injury.

Considering these reports, IL-6 may be related to the formal pathogenesis of OA, suggesting that active cartilage destruction occurs at the greatest rate in earlier KL grades. In other words, late-stage KL grading may indicate the "burnt ruins" acquired after active inflammation where IL-6 is more directly involved than TNFα.

### Correlation between cytokine concentrations and the WOMAC score

The present study demonstrated that TNFα is significantly correlated with the WOMAC score including the subscores. However, IL-6 was not correlated with the WOMAC score excluding the subscore of stiffness, which indicates that IL-6 primarily affects the progress of the degeneration of joint cartilage in OA that leads to joint stiffness.

Furthermore, we found a correlation only with the subscore of stiffness, which can be derived from the constructive degradation of the cartilage, and we found no correlation with the subscore of pain. A previous paper reported a negative correlation between IL-6 activity and radiographic OA scoring in dog OA models [20], and this coincides with the results of the present study.

The present study has some limitations. First, we did not examine the gene expression of each cytokine. Variations in several genes that regulate inflammation have been reported to be associated with the differential expression of inflammatory mediators [37-39], some of which have been associated with OA pathology [40-44]. Thus, further studies including genetic investigations are needed, particularly for NGF. Second, the obscurity of KL grading, which is based on an unclear definition of the joint space findings, could have affected the results of the present study. Precise evaluation using more quantified grading systems such as the OARSI atlas should be performed in future studies. Third, we could not examine normal knees because it may be technically difficult and ethically improper to obtain control synovial fluid from intact knee joints. We should evaluate knees with other injuries or degradations in future studies. Last, we only examined the synovial fluid. It will be important to assess the serum levels of these cytokines and compare them with both the levels of cytokines in the synovial fluid and the grading scores.

## Conclusions

The present study demonstrated that the concentrations of proinflammatory cytokines can be correlated with the KL grades and WOMAC scores of knee OA patients. TNFα did not have a significant correlation with the radiographic grading, while it did with the WOMAC scoring. IL-6 had a significant negative correlation with KL grading, and only a weakly significant correlation with the subscore of stiffness. These 2 cytokines were moderately correlated, and the results suggest that these cytokines play a role in the pathogenesis of synovitis in osteoarthritic knees in different ways. TNFα is correlated with pain, whereas IL-6 is correlated with joint function. NGF was undetectable in the synovial fluid, illustrating the need for differently designed experiments in future studies.

## Competing interests

The authors declare that they have no competing interests.

## Authors' contributions

SO designed and performed all the experiments, analyzed data, and drafted the paper. TK, TM, MM, GI, GA, TI, EH, KY, MY, and YE harvested synovial fluid in their outpatient clinic and performed experiments. TT, KT, and SO supervised the project and edited the manuscript. All authors contributed to data interpretation and have read and approved the final manuscript.

## Pre-publication history

The pre-publication history for this paper can be accessed here:

http://www.biomedcentral.com/1471-2474/12/144/prepub
